# Metformin in prevention and treatment of antipsychotic induced weight gain: a systematic review and meta-analysis

**DOI:** 10.1186/s12888-016-1049-5

**Published:** 2016-10-03

**Authors:** Varuni Asanka de Silva, Chathurie Suraweera, Suhashini S. Ratnatunga, Madhubashinee Dayabandara, Nimali Wanniarachchi, Raveen Hanwella

**Affiliations:** 1 Faculty of Medicine, University of Colombo, Colombo, Sri Lanka; 2 University Psychiatry Unit, National Hospital of Sri Lanka, Colombo, Sri Lanka

## Abstract

**Background:**

Most antipsychotics are associated with weight gain and other metabolic complications. Several randomized trials have shown metformin to be effective, but this still hasn’t been included in clinical guidelines on managing antipsychotic induced weight gain.

**Methods:**

All double blind placebo controlled trials assessing the efficacy of metformin in the treatment of antipsychotic induced weight gain were included. Cochrane Central Register of Controlled Trials (CENTRAL) and MEDLINE were searched for the period January 2000-December 2015. Meta-analysis was carried out using the random effects model.

**Results:**

Meta analysis of 12 published studies with a total of 743 patients found that in patients treated with antipsychotics, metformin treatment resulted in significantly better anthropometric and metabolic parameters than placebo. The mean change in weight was −3.27 kg (95 % CI −4.66 to −1.89) (*Z* = 4.64, *p* < 0.001). Metformin compared to placebo resulted in significant reduction in BMI [−1.13 kg/m^2^ (95 % CI −1.61 to −0.66)] and insulin resistance index [−1.49 (95 % CI −2.40 to −0.59)] but not fasting blood sugar [−2.48 mg/dl (95 % CI −5.54 to 0.57].

**Conclusion:**

This meta-analysis confirms that metformin is effective in treating antipsychotic induced weight gain in patients with schizophrenia or schizoaffective disorder.

**Electronic supplementary material:**

The online version of this article (doi:10.1186/s12888-016-1049-5) contains supplementary material, which is available to authorized users.

## Background

Most antipsychotics are associated with weight gain and other metabolic complications [1]. Prevalence of metabolic syndrome is higher in patients treated with antipsychotics than in drug naive patients with schizophrenia. Metabolic syndrome is more likely with second generation antipsychotics than first generation antipsychotics [2]. Rate of weight gain is highest in the first six months after commencing treatment however patients continue to gain weight during the course of treatment [3]. Clozapine and olanzapine have the highest risk of weight gain while aripiprazole, lurasidone and ziprasidone have the lowest risk [4–6].

The standardized mortality ratio in schizophrenia is 1.5 times that of the general population [7]. This risk has been increasing over the recent past [8]. Some of this increased risk is attributed to the use of second generation antipsychotics [9]. Coronary heart disease is the major cause of death in patients with schizophrenia. Increased rates of cigarette smoking, obesity and metabolic syndrome caused by life style factors and side effects are major contributors [10]. The beneficial effects of better compliance with medication and reduced suicide rates due to second generation antipsychotics are offset by the deaths due to antipsychotic induced weight gain [9].

Behavioural interventions consisting of life style modifications are effective in reducing antipsychotic induced weight gain [11]. These can be used alone or as an adjunctive to pharmacological treatment. There is no significant difference between the types of interventions of individual or group therapy and nutritional counselling and cognitive behaviour therapy [11].

Metformin enhances the action of insulin in the liver and thereby decreases the rate of hepatic glucose production [12]. Metformin also increases peripheral utilization and suppresses appetite [13]. Metformin is recommended as first line treatment in type 2 diabetes mellitus [14]. It is also used to treat obesity is non diabetics. Metformin may contribute to weight reduction in the obese by reducing insulin resistance and by suppressing appetite. However its efficacy in treating obesity in non-diabetics has not been established [15].

Although several studies have shown metformin to be effective, this still hasn’t been included in clinical guidelines on managing antipsychotic induced weight gain. Several RCT which evaluated the efficacy of metformin in treating antipsychotic induced weight gain have been published recently [16, 17]. Therefore it is important that the evidence regarding metformin is synthesized.

The objective of this review was to assess the efficacy of metformin for treating or preventing antipsychotic induced weight gain in patients with schizophrenia or schizoaffective disorder.

## Methods

A study protocol was developed and the meta analysis was conducted according to it. The study protocol is available from the corresponding author on request. The meta-analysis was conducted based on the Preferred Reporting Items for Systematic Reviews and Meta-Analyses (PRISMA) [18].

### Types of studies

All published, randomized controlled trials with double blind assessment of outcome were included in the review. Open label trials and observational studies were excluded.

### Participant characteristics and diagnosis

Participants of both sexes and all age groups with schizophrenia or schizoaffective disorder diagnosed according to DSM IV, DSM-5 or ICD-10 criteria treated with antipsychotics were included [19–21].

### Types of interventions

All double blind placebo controlled trials assessing the efficacy of metformin in the treatment of antipsychotic induced weight gain were included. Trials which tested a combination of metformin with lifestyle modification for weight gain as an adjunct were also included.

### Outcome measures

The primary outcome measure of efficacy was mean change in weight in kg between pre-treatment and end of study weight. Secondary outcome measures were change in BMI (kg/m^2^), fasting blood sugar (mg/dl) and insulin resistance index (IRI). The principal summary measure used was mean difference.

### Search methods for identification of studies

A study protocol detailing sources of data, search strategy, outcome measures, study selection criteria and statistical analysis was developed. Cochrane Central Register of Controlled Trials (CENTRAL) MEDLINE and EMBASE were searched for the period January 2000-December 2015. We also looked at the references of selected full text articles. We used the following search terms. Randomized controlled trial OR randomized OR clinical trial OR randomized controlled trial AND metformin AND antipsychotic agents OR dopamine antagonists OR atypical antipsychotics OR antipsychotic induced weight gain OR second generation antipsychotics OR olanzapine OR clozapine OR risperidone OR aripiprazole OR ziprasidone OR quetiapine.

### Data collection process

Data was extracted from studies independently using a data collection form by two investigators (VdeS and R.H.). Disagreements were resolved by a third investigator (C.S.).

### Risk of bias

Methodological quality of the included studies was evaluated using the Cochrane risk of bias tool and the Jadad scale (Additional file 1: Table S1) [22]. Sequence generation, allocation concealment, blinding of participants, personnel and outcome assessors, incomplete outcome data, selective outcome reporting, and other potential sources of bias were assessed according to these tools.

### Statistical analysis

Statistical analysis was carried out using Review Manager version 5.2 [23]. Meta-analysis was carried out using the random effects model of DerSimonian and Laird because the subjects and interventions in the studies have differed in ways that would have impacted on the results [24]. The presence of heterogeneity between studies was tested using the Cochran’s Q. The magnitude of heterogeneity was determined using the I^2^ statistic. We analysed the mean change in body weight, BMI, fasting glucose, insulin resistance and percentage change of body weight. Six studies reported the change in body weight percentage [16, 17, 25–28]. In other studies percentage was derived by dividing the change in weight by the mean baseline weight. When data on standard deviations were missing, it was calculated using the standard error of subgroups or confidence intervals. Funnel plot analysis was used to detect publication bias using Begg–Mazumdar method and Egger’s test. This was done using the software Comprehensive meta analysis (trial version) [29].

### Subgroup analysis

Subgroup analysis was carried out for weight and BMI comparing adults and children and also first episode versus chronic patients.

### Sensitivity analysis

Sensitivity analysis was carried out by excluding the studies carried out in Chinese populations.

### Ethical issues

Ethics clearance was not sought and consent was not obtained as this is a secondary analysis of published data and does not contain any individual clinical data.

## Results

### Study selection

**Fig. 1:**
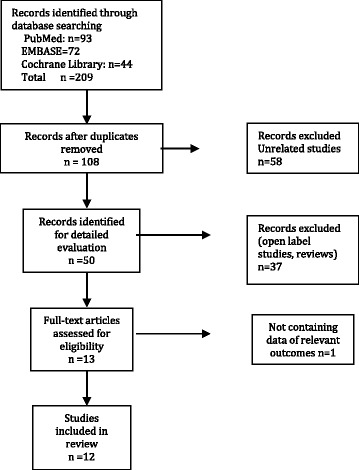
Study flow summary

A total of 137 studies were screened. Thirteen RCTs were identified but one was excluded cause it did not contain adequate data on the primary outcome [20, 30]. Twelve studies of adults and children were included in the analysis (Fig. 1).

### Description of studies

All were parallel group randomised controlled trials comparing treatment with metformin or placebo of patients on atypical antipsychotics (Table 1). A total of 743 participants were included. In five studies patients were treated with a specific antipsychotic (olanzapine, risperidone or clozapine) [31–35]. Four studies were conducted in China, one in Taiwan, three in Venezuela, two in the United States and one each in Saudi Arabia and Sri Lanka. Sample size ranged from 16–75 in each group. Two studies included children and adolescents [25, 36]. One included only female patients who had amenorrhoea [26]. Five studies included patients who had gained more than 7–10 % of their body weight after commencing treatment with antipsychotics [16, 27, 28, 34, 36]. In 5 studies mean pre-treatment BMI was ≥25 [16, 17, 32, 35, 36]. Five studies were conducted in first episode patients [25–28, 34]. In most studies the dose of metformin was 1000 mg a day.

**Table 1:** Study Characteristics

	Study	Methods	Participants	Country	Numbers	Intervention
1.	Armen 2008	Parallel group RCT Duration 12 weeks	Age < 20 years On risperidone 2–6 mg	Saudi Arabia	Metformin *N* = 16 Placebo *N* = 16	Metformin 500 mg twice daily or placebo
2.	Baptista 2006	Parallel group RCT Duration 14 weeks	Age ≥18 years Olanzapine monotherapy > 4 months	Venezuela	Metformin *N* = 19 Placebo *N* = 18	Metformin 850–1750 mg Balanced diet of 2500-300Kcal
3.	Baptista 2007	Parallel group RCT Duration 12 weeks	Age ≥18 years Olanzapine monotherapy > 4 months	Venezuela	Metformin *N* = 36 Placebo *N* = 36	Metformin 850–2550 mg or placebo Diet and exercise counseling at start of study
4.	Carrizo 2009	Parallel group RCT Duration 14 weeks	clozapine treatment > 3 months	Venezuela	Metformin *N* = 31 Placebo *N* = 30	Extended release metformin 500–1000 mg/day or placebo
5.	Chen	Parallel group RCT Duration 24 weeks	clozapine treatment > 3 months BMI ≥24 or one metabolic syndrome criteria	Taiwan	Metformin *N* = 28 Placebo *N* = 27	Metformin 1500 mg/day
6.	De Silva 2015	Parallel group RCT Duration 24 weeks	Age ≥18 years Weight gain > 10 % of body weight Females 78.8 %	Sri Lanka	Metformin *N* = 34 Placebo *N* = 32	Metformin or placebo 500 mg twice daily Diet and exercise counseling given at start of study
7.	Jarskog 2013	Parallel group RCT Duration 16 weeks	Age 18–65 years BMI ≥27 Duration of illness ≥ 1 year Females 30.8 %	United States	Metformin *N* = 75 Placebo *N* = 71	Metformin 500 mg twice daily increased upto maximum of 2000 mg/day or placebo Weekly diet and exercise counseling
8.	Klein	Parallel group RCT Duration 16 weeks	Age 10–17 years Gained > 10 % body weight	United States	Metformin *N* = 34 Placebo *N* = 32	Metformin 850 mg twice daily or placebo nutritional counseling
9.	Wang 2012	Parallel group RCT Duration 12 weeks	Age 18–60 years Gained > 7 % of body weight	China	Metformin *N* = 32 Placebo *N* = 34	Metformin 500 mg twice daily or placebo
10.	Wu 2012	Parallel group RCT Duration 24 weeks	Age 18–40 years First episode Female patients only	China	Metformin *N* = 42 Placebo *N* = 42	Metformin 1000 mg/day or placebo
11.	Wu 2008a JAMA	Parallel group RCT Duration 12 weeks	Age 18–45 years First episode patient who gained > 10 % of bodyweight	China	Metformin *N* = 32 Placebo *N* = 32	Metformin 750 mg or placebo (also metformin + lifestyle and lifestyle + placebo groups)
12.	Wu 2008b AM J	Parallel group RCT Duration 12 weeks	Age 18–50 years First episode patients on olanzapine	China	Metformin *N* = 18 Placebo *N* = 19	Metformin 250 mg thrice daily or placebo No special diet or exercise program

### Risk of bias

Most information was from studies at low risk of bias. The detailed risk of bias table is given as a additional file (Additional file 1: Table S1). Quality of trials was also assessed using the Jadad scale. One trial scored 2 and the others scored between 3–5 (Additional file 1: Table S1).

### Synthesis of results

#### Body weight

**Fig. 2:**
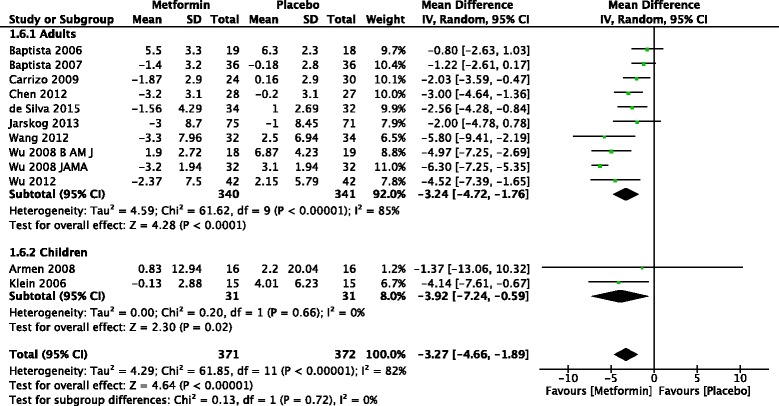
Forest plot of difference of mean weight change of metformin versus placebo

The forest plot from the meta analysis weight change is given in Fig. 2. Ten studies of adults and two of children were included in the meta analysis of weight. Of them seven studies in adults and one in children showed that there was significant difference in weight gain between metformin and placebo. Meta-analysis of 12 studies found that that treatment with metformin resulted in significantly more weight loss than placebo in patients treated with antipsychotics [−3.27 kg (95 % CI −4.66 to −1.89) (*Z* = 4.64, *p* < 0.001).

Meta-analysis of the studies found that that the percentage of body weight loss with metformin was significantly more than with placebo [−5.07 (95 % CI −6.67 to −3.45) (*Z* = 6.13, *p* < 0.001).

#### Body mass index

**Fig. 3:**
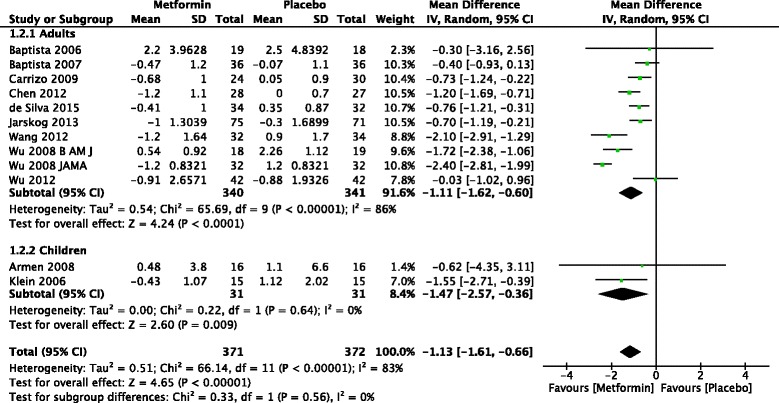
Forest plot of mean change in BMI in patients treated with metformin versus placebo

The forest plot from the meta analysis of change in BMI is given in Fig. 3. Ten studies of adults and two of children were included in the meta analysis of BMI. Of them seven studies in adults and one in children showed that there was significant difference in BMI between metformin and placebo. Meta-analysis of 12 studies found that that treatment with metformin resulted in significantly more reduction in BMI than placebo in patients treated with antipsychotics [−1.13 kg/m^2^ (95 % CI −1.61 to −0.66)] (*Z* = 4.65, *p* < 0.001).

#### Fasting blood sugar

**Fig. 4:**
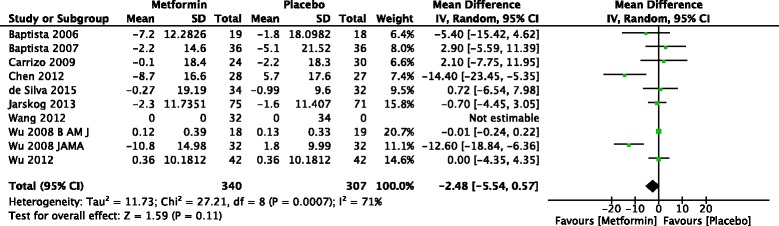
Forest plot of mean change in fasting blood sugar in patients treated with metformin versus placebo

The forest plot from the meta analysis of change in FBS is given in Fig. 4. Ten studies of adults were included and two studies found a significant difference between metformin and placebo. Meta-analysis of 10 studies found that that treatment with metformin did not result in significant reduction in FBS compared to placebo in patients treated with antipsychotics [−2.48 mg/dl (95 % CI −5.54 to 0.57)] (*Z* = 1.59, *p* = 0.11).

#### Insulin resistance index

**Fig. 5:**
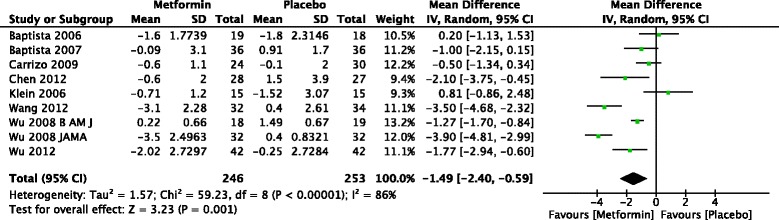
Forest plot of mean change in insulin resistant index in patients treated with metformin versus placebo

The forest plot from the meta analysis of change in insulin resistance index is given in Fig. 5. Nine studies in adults reported change in IRI. One study of 16 weeks duration gave data only for the week 8 value [36]. Five studies reported a significant difference between metformin and placebo. Meta-analysis of 9 studies found that that treatment with metformin resulted in significant reduction in IRI than placebo in patients treated with antipsychotics [−1.49 (95 % CI −2.40 to −0.59) (*Z* = 3.23, *p* < 0.001)].

### Discontinuation and adverse events

Discontinuation was reported in 5 trials. De Silva et al. reported one discontinuation due to dizziness in metformin group and 3 due to development of diabetes in placebo group [16]. Jarskorg et al. reported 11 in the metformin and 8 on placebo discontinued to intolerability [17]. Wu et al. reported that 1 in metformin group and 2 in placebo withdrew due to psychosis [26]. Wang et al. reported 3 discontinuations due to nausea and psychosis [27]. Wu et al. reported 5 discontinuations due to psychosis [28]. Only one trial reported diarrhoea was significantly more in the metformin group compared to placebo [17]. Three trials reported no significant difference in moderate adverse events [26–28].

### Sub group analysis

#### Adults versus children

Subgroup analysis was carried out for weight and BMI comparing adults and children. There was significant mean difference in weight favouring metformin in both adults [−3.24 (95 % CI −4.72 to −1.76) (*Z* = 4.28, *p* < 0.001)] and children [−3.92 (95 % CI −7.24 to −0.59) (*Z* =2.30, *p* = 0.02)].

There was significant difference in change in BMI in adults −1.11 (95 % CI −1.62 to −0.60) (Z = 4.24, *p* < 0.001)] and children [−1.47 (95 % CI −2.57 to −0.36) (*Z* = 2.6, *p* = 0.009)].

### First episode versus chronic illness

**Fig. 6:**
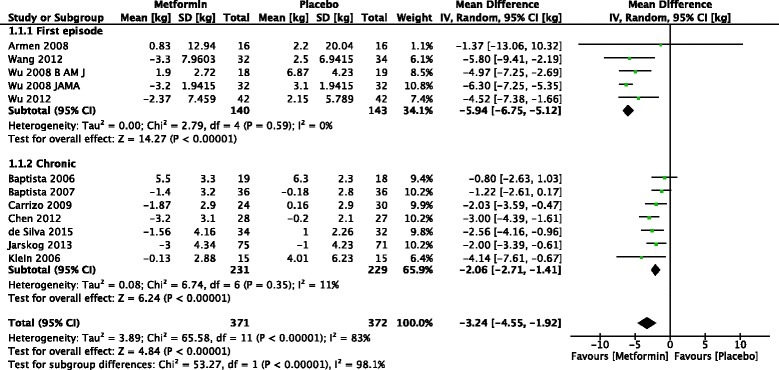
Forest plot of subgroup analysis of weight change in first episode versus chronic patients

Subgroup analysis shows that the five trials which included first episode patients −5.94 kg (95 % CI 6.75 to −5.12) showed a much larger difference in mean body weight change than trials of chronic patients −2.06 kg (95 % CI −2.71 to −1.41) (Fig. 6).

### Sensitivity analysis

**Fig. 7:**
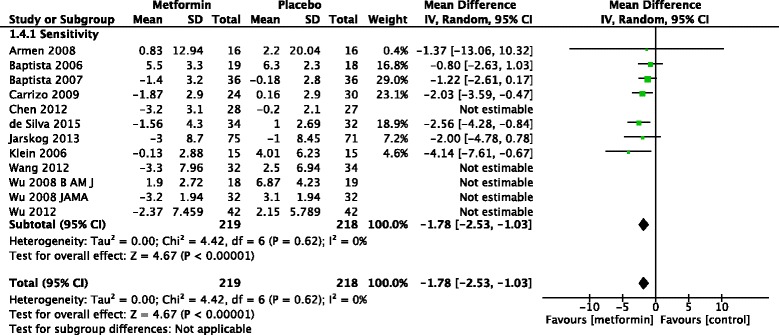
Sensitivity analysis excluding ethnic Chinese patients

Because the largest difference in body weight was in the ethnic Chinese population we conducted a sensitivity analysis by excluding these studies (Fig. 7). Metformin was significantly more effective than placebo even after excluding these studies (*Z* = 4.67, *p* < 0.001).

### Publication bias

**Fig. 8:**
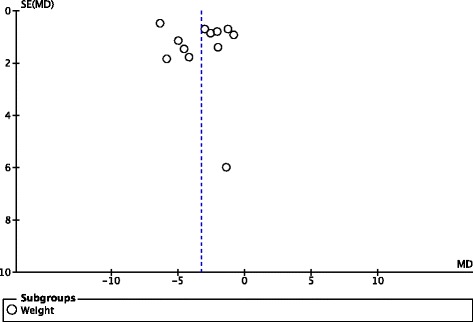
Funnel plot metformin versus placebo for treatment of antipsychotic induced weight gain

Funnel plot with standard error on the vertical axis and treatment effect on the horizontal axis is given in Fig. 8. Publication bias was assessed using Begg–Mazumdar method and Egger’s test. There was no evidence of asymmetry of treatment effect for weight (Begg Mazumdar: Kendall’s *t* = −0.27, *P* =0.217; Egger’s test *p* = 0.662).

### Heterogeneity

There was significant heterogeneity among the studies (*P* < 0.001, I2 = 84 %). Subgroup analysis found that there was significant heterogeneity in the adult studies (*P* < 0.001, I2 = 87 %) but not studies of children (*P* = 0.66, I2 = 0 %).

## Discussion

Meta analysis of 12 published studies with a total of 743 patients found that in patients treated with antipsychotics, metformin treatment resulted in significantly better anthropometric and metabolic parameters than placebo. The mean difference in weight was −3.27 kg (95 % CI −4.66 to −1.89) (*Z* = 4.64, *p* < 0.001). Metformin compared to placebo resulted in significant reduction in BMI [−1.13 kg/m^2^ (95 % CI −1.61 to −0.60)] and insulin resistance index [−1.49 (95 % CI −2.40 to −0.59)] but not fasting blood sugar [−2.48 mg/dl (95 % CI −5.54 to 0.57)].

Although pooled data shows that the mean weight loss is −3.27 kg it is important to know if this is clinically meaningful. Weight losses of 5 % or more can result in clinically significant reduction of morbidity and mortality [37]. Wang et al. reported that 40.6 % in the metformin treated group and 7 % in the placebo group reduced their body weight by 7 % [27]. Wu et al. reported that only 16.7 % in the metformin group gained > 7 % of their body weight compared to placebo group (63.16 %). Thus is appears that the metformin results in clinically significant weight loss in about half the patients.

Publication bias occurs when studies with small difference between intervention and control groups or those showing no significant difference between the two medications are less likely to be accepted for publication. Funnel plot analysis showed there was no significant publication bias.

Heterogeneity occurs when there is variation in true effect size. This variation can occur due to methodological differences in the type of participants, interventions and outcome measures between clinical trials. The studies included in this meta-analysis had a wide variation in patient characteristics. We pooled together studies which included first episode as well as chronic patients, those who had gained more than 10 % of the bodyweight, those who were commencing treatment with atypical antipsychotics, children and adults and patients of different ethnic origin. We found significant heterogeneity among the studies. Therefore we used a random effects model to analyse the data. Subgroup analysis showed that most of the heterogeneity was due to the pooling of studies of first episode patients with chronic patients.

Metformin appears to be more effective in preventing antipsychotic induced weight gain in first episode patients than in chronic patients who have already gained weight. Subgroup analysis shows that the pooled mean difference in weight of the five trials which included first episode patients −5.94 kg (95 % CI 6.75 to −5.12) was much larger than that of trials of chronic patients −2.06 kg (95 % CI −2.71 to −1.41). This could be due to the metabolic changes which occur with continued use of antipsychotics. Antipsychotic naïve patients show rapid and continuous weight gain in the first few weeks. During the first 12 weeks mean weight gain is about 3.8 kg with a 1 point increase in BMI [38]. This weight gain continues throughout the duration of antipsychotic treatment. With continuous weight gain insulin resistance increases. A study which followed up antipsychotic naïve patients treated with second generation antipsychotics over 8 weeks reported that serum insulin decreased at week 2, returned to baseline at week 4, and increased at week 8 [39]. In patients treated over a long period insulin resistance increases with time. Metformin may be more effective in preventing weight gain before the onset of significant insulin resistance and thus shows more efficacy in antipsychotic naïve patients. Once these metabolic changes have occurred metformin may be less effective in preventing or reversing weight gain.

Sub group analysis shows that metformin is effective in children There were only two studies conducted in children and adolescents [25, 36]. Both were small studies with 15 or 16 participants in each arm. One study included participants aged 10–17 years and the other study included children with a mean age of 8.9 years and 11.25 years. Out of the two studies only one showed significant difference in weight change. In this study the placebo group gained a mean of 4.01 kg (SD 6.23) of weight while the metformin group lost 0.13 kg. There are a few open label studies too which found that metformin was effective in treating weight gain in children on antipsychotics [40, 41]. How ever all these studies are small and the evidence for the use of metformin in children is not as robust as in adults.

Because overall mean difference in weight was much larger in Chinese patients compared to non-Chinese patients we conducted a sensitivity analysis excluding these studies. Sensitivity analysis shows that excluding trials conducted in Chinese populations did not significantly change the outcome. The non Chinese RCTs were conducted among Hispanic, Caucasian and South Asian populations. We also found that that of the five studies conducted in ethnic Chinese patients four included first episode patients. Therefore the larger mean difference in weight in ethnic Chinese could be due to genetic effects or because the studies were of first episode patients who respond better than chronic patients.

Only 2 RCTs were of 6 months duration [16, 26]. Two trials were of 16 weeks duration [17, 36]. The other trials were of 12–14 weeks duration. The trials that were longer than 12 weeks showed that patients on metformin continued to lose weight with time. Therefore it is likely that continuing metformin is beneficial. Since the data from trials is limited to 6 months it is not known if the weight loss continues, plateaus or if there is reversal after that period.

Only one RCT included an arm of lifestyle modification [28]. It found that metformin plus life style modification was superior to metformin treatment alone. A meta-analysis of nonpharmacological interventions also found that significant nonpharmacological intervention such as dietary counseling and cognitive behaviour therapy were more effective than treatment as usual in reducing antipsychotic induced weight gain [11]. This meta analysis which included trials using cognitive behavior therapy, nutritional counseling or combined nutritional and exercise interventions reported a weighted mean difference of −2.56 kg (95 % CI −3.20 to −1.92) favouring the intervention. This is similar to the mean difference in weight achieved in metformin trials [11].

Several meta analysis have been conducted previously. Meta-analysis by Mizuno et al. and Maayan et al. analysed several pharmacological interventions including metformin. Bergman et al. included 7 RCT and Praharaj et al. four. All these meta-analysis included less RCTs than our study. However all reported that metformin significantly reduced weight and other anthropometric measures [42]. The most comprehensive is the one by Zhen et al. which included 21 RCTS including 10 trials published in the Chinese language which were not included in our study. The 13 trials conducted among Chinese populations reported a pooled standardised mean difference of −0.69 compared to −0.40 in trials conducted in non-Chinese. However this meta analysis used values at the end of follow up period instead of the difference between end and baseline values the end of follow up period instead of the difference between end and baseline values for waist circumference and fasting blood sugar.

There are several limitation to this meta-analysis. There was significant heterogeneity across the studies. Sub group analysis showed that this was probably due to pooling of trails conducted in first episode and chronic patients. We did not include unpublished data and results of several trials published in the Chinese language.

## Conclusion

This meta-analysis confirms that metformin is effective in treating antipsychotic induced weight gain in patients with schizophrenia or schizoaffective disorder. This meta analysis which included recently published data showed a larger mean difference in weight than that reported in previous meta analysis. There is sufficient evidence to recommend commencing metformin in patients with antipsychotic induced weight gain. Considering the magnitude of effect in patients with first episode psychosis we recommend that metformin is commenced in all patients who show evidence of weight gain.

### Recommendations

Both adults and children receiving antipsychotics should be monitored for weight gain and other metabolic complications as this will allow early intervention. All patients should be provided advice on diet and other life style modifications. Switching to an antipsychotic with less risk of weight gain is known to be beneficial. We recommend that clinicians consider prescribing metformin for patients when the above strategies are not adequate to control weight gain.

## Additional file

Additional file 1: Table S1. Cochrane Risk of Bias assessment. Assessment of risk of bias of included studies. (DOC 58 kb)

## Abbreviations

CI: Confidence interval
DSM-IV: Diagnostic and Statistical Manual-IV
ICD-10: International Classification of Mental and Behavioural Disorders
RCT: Randomized control trial
SD: standard deviation
