# Supplementary material for: Metformin in prevention and treatment of antipsychotic induced weight gain: a systematic review and meta-analysis
Source: BMC Psychiatry. 2016 Oct 3;16:341. doi: 10.1186/s12888-016-1049-5 (PMC5048618; doi:10.1186/s12888-016-1049-5)
Supplement: Additional file 1: Table S1. — Cochrane Risk of Bias assessment. Assessment of risk of bias of included studies. (DOC 58 kb) [file 12888_2016_1049_MOESM1_ESM.doc]

**Supplementary Table 1. Cochrane Risk of Bias assessment**

|  | Assessments for sequence generation | Allocation sequence concealment | Blinding of participants and personnel | Incomplete outcome data | Selective outcome reporting |
| --- | --- | --- | --- | --- | --- |
| Armen 2008 | Unclear risk | Unclear risk | Low risk | High Risk | High risk |
| Baptista 2006 | Low risk | Unclear risk | Unclear risk | Low risk | Low risk |
| Baptista 2007 | Low risk | Low risk | Low risk | Unclear risk | Low risk |
| Carrizo 2009 | Low risk | Low risk | Low risk | Low risk | Low risk |
| Chen 2012 | Unclear risk | Unclear risk | Low risk | Low risk | Unclear risk |
| De Silva 2015 | Low risk | Low risk | Low risk | Low risk | Low risk |
| Hebrani 2015 | Unclear risk | Unclear risk | Unclear risk | High risk | Low risk |
| Jarskog 2013 | Unclear risk | Low risk | Unclear risk | Low risk | Low risk |
| Klein 2006 | Unclear risk | Unclear risk | Low risk | Low risk | Low risk |
| Wang 2012 | Unclear risk | Unclear risk | Unclear risk | Low risk | Low risk |
| Wu 2008 a AmJ | Low risk | Low risk | Low risk | Low risk | Low risk |
| Wu 2008 b | Low risk | Low risk | Low risk | Low risk | Low risk |
| Wu 2012 | Low risk | Low risk | Unclear risk | Low risk | Low risk |

**Supplementary Table 2. Assessing the quality of trials using the Jadad scale**

|  | Randomised | Method used to randomise | Described as double blind | Method of double blind | Withdrawal and dropout | Total |
| --- | --- | --- | --- | --- | --- | --- |
| Armen 2008 | 1 | 0 | 1 | 0 | 0 | 2 |
| Baptista 2006 | 1 | 1 | 1 | 0 | 0 | 3 |
| Baptista 2007 | 1 | 1 | 1 | 0 | 0 | 3 |
| Carrizo 2009 | 1 | 1 | 1 | 0 | 0 | 3 |
| Chen 2012 | 1 | 0 | 1 | 1 | 1 | 4 |
| De Silva 2015 | 1 | 1 | 1 | 1 | 1 | 5 |
| Jarskog 2013 | 1 | 1 | 1 | 1 | 1 | 5 |
| Klein 2006 | 1 | 0 | 1 | 1 | 0 | 3 |
| Wang 2012 | 1 | 0 | 1 | 0 | 1 | 3 |
| Wu 2008 a AmJ | 1 | 1 | 1 | 1 | 1 | 4 |
| Wu 2008 b | 1 | 1 | 1 | 0 | 1 | 4 |
| Wu 2012 | 1 | 1 | 1 | 0 | 1 | 4 |
